# Prognostic Value of ^99m^Tc-Pertechnetate Thyroid Scintigraphy in Radioiodine Therapy in a Cohort of Chinese Graves' Disease Patients: A Pilot Clinical Study

**DOI:** 10.1155/2015/974689

**Published:** 2015-03-24

**Authors:** Haifeng Hou, Shu Hu, Rong Fan, Wen Sun, Xiaofei Zhang, Mei Tian

**Affiliations:** ^1^Department of Nuclear Medicine and PET Center, The Second Affiliated Hospital of Zhejiang University School of Medicine, 88 Jiefang Road, Hangzhou, Zhejiang 310009, China; ^2^Zhejiang University Medical PET Center, Hangzhou, Zhejiang 310009, China; ^3^Institutes of Nuclear Medicine and Molecular Imaging, Zhejiang University, Hangzhou, Zhejiang 310009, China; ^4^Key Laboratory of Medical Molecular Imaging of Zhejiang Province, Hangzhou, Zhejiang 310009, China; ^5^Department of Nuclear Medicine, Peking University Shenzhen Hospital, Shenzhen, Guangdong 518036, China; ^6^Department of Clinical Epidemiology and Biostatistics, The Second Affiliated Hospital of Zhejiang University School of Medicine, Hangzhou, Zhejiang 310009, China

## Abstract

*Objectives*. This study is to assess the prognostic value of ^99m^Tc-pertechnetate thyroid scintigraphy for predicting the outcomes of fixed low dose of radioiodine therapy (RIT) in a cohort of Chinese Graves' disease (GD) patients. *Materials and Methods*. This is a retrospective study of GD patients who received RIT with a single dose of radioiodine (5 mCi). All the patients received ^99m^Tc-pertechnetate thyroid scintigraphy prior to RIT. Thyroid mass, ^99m^Tc-pertechnetate uptake, gender, age at diagnosis, duration of the disease, ophthalmopathy, and serum levels of FT4, FT3, TT4, and TT3 prior to RIT were analyzed as potential interference factors for outcomes of RIT. *Results*. One hundred and eighteen GD patients who completed RIT were followed up for 12 months. The outcomes (euthyroidism, hypothyroidism, and hyperthyroidism) were found to be significantly associated with thyroid mass and ^99m^Tc-pertechnetate uptake. Patients with thyroid mass ≤ 40.1 g or ^99m^Tc-pertechnetate uptake ≤ 15.2% had higher treatment success. *Conclusions*. A fixed low dose of 5 mCi radioiodine seems to be practical and effective for the treatment of Chinese GD patients with thyroid mass ≤ 40.1 g and ^99m^Tc-pertechnetate uptake ≤ 15.2%. This study demonstrates ^99m^Tc-pertechnetate thyroid scintigraphy is an important prognostic factor for predicting the outcomes of RIT.

## 1. Introduction

Radioiodine therapy (RIT) is considered to be the effective treatment for Graves' disease (GD); however, the optimal radioiodine dose (administered activity) in the treatment of GD remains unsettled [1]. Although it is generally accepted that cure rates of GD may increase with the radioiodine dose, higher dose of radioiodine might cause unnecessary radiation exposure. Elevated risk of cancers (especially cancer of the stomach, kidney, and breast) was found in association with the cumulative increase of radioiodine dose [2].

In addition to controversial results on the optimal dose of radioiodine administered, consensus on the predictive factors for RIT is still missing among the radionuclide therapy communities. Previously, age, gender, clinical symptoms, thyroid mass, serum TSH receptor antibodies (TRAb) levels, thyroid uptake, and history of thyrostatic drugs use have been reported to be correlated with the success rates of RIT [1]. However, until now, the response to RIT in patients with GD remains unpredictable, and factors postulated to predict the outcomes have not been proved clinically useful or widely adopted in clinical practice.

In the present study, we used a fixed comparatively low dose radioiodine (5 mCi) and reported the value of ^99m^Tc-pertechnetate thyroid scintigraphy for predicting the outcomes of RIT in a cohort of Chinese GD patients.

## 2. Patients and Methods

### 2.1. Patients

One hundred and twenty-eight GD patients treated with a fixed dose of radioiodine (5 mCi) in the Nuclear Medicine Clinic were retrospectively evaluated from January, 2011, to June, 2012. All patients came from iodine-sufficient areas in the East China. Among all the patients, 80 were initially treated with methimazole (MMI) or propylthiouracil (PTU) for at least 12 months but still had hyperthyroidism after withdrawal or reducing the medication dosage. Before being referred to RIT, the antithyroid drugs (ATDs) treated GD patients remained medication free for at least 1 month. The other 48 patients had not taken ATDs because of self-selection or significant side effect (agranulocytosis, posttherapy liver failure or allergy). All the patients received RIT with a fixed dose of 5 mCi radioiodine at the Nuclear Medicine Clinic and signed the written informed consents. This study has been approved by the local ethics committee.

Patients were followed up for at least 12 months after RIT. RIT was considered successful if euthyroidism or hypothyroidism was achieved, which was diagnosed on the basis of thyroid function tests. RIT was considered as a failure, when the patients remained hyperthyroid, had to use ATDs, or received a second dose of RIT before the end of the followup. Ten patients were excluded from the data analysis due to the failure of follow-up.

The gender, age at diagnosis, duration of the disease, thyroid mass, ^99m^Tc-pertechnetate uptake, ophthalmopathy, and serum levels of FT4, FT3, TT4, and TT3 prior to RIT were studied as potential interference factors for RIT.

### 2.2. Thyroid Scintigraphy

All GD patients were instructed to follow a low iodine diet and avoid iodine-rich products for 15 days prior to thyroid scintigraphy. Thyroid scan and uptake were performed at 20 min after intravenous injection of 185 MBq (5 mCi) of ^99m^Tc-pertechnetate (Atom High Tech Co., Ltd., Shanghai, China). A SPECT scintillation camera equipped with a low-energy, high-resolution, and parallel-hole collimator (E.CAM, Siemens Medical Solutions) was used for thyroid scintigraphic scan. The method for the calculation of thyroid mass and ^99m^Tc-pertechnetate uptake was previously described by Ramos et al. [3].

### 2.3. Statistical Analysis

All statistical analyses were carried out using SPSS version 16.0 for Windows (SPSS Inc., Chicago, IL, USA). Data were expressed as mean ± standard deviation (SD). The chi-squared test and Mann-Whitney *U* test were performed to compare variables in the two groups (hypothyroid and euthyroid versus hyperthyroid patients). Logistic regression analysis was used to identify the associated factors with RIT success (euthyroidism and hypothyroidism). Stepwise method was used for variables selection. The receiver operator characteristic (ROC) curve was used to identify the optimal threshold for thyroid mass and ^99m^Tc-pertechnetate uptake to discriminate RIT success or failure (persistence of hyperthyroidism) of RIT. The significance level was set at 5%.

## 3. Results

### 3.1. Patients and Outcomes after RIT

Baseline, pre-RIT patient laboratory and clinical characteristics are listed in Table 1. A total of 118 patients treated by RIT were included in this study, of which 49 patients (41.53%) were euthyroid, 35 patients (29.66%) were hypothyroid, and 34 patients (28.81%) remained hyperthyroid. Patients were followed up after RIT for at least 12 months.

### 3.2. Comparative Analysis between the Variables and the Outcomes after RIT

Thyroid mass (*P* < 0.001), ^99m^Tc-pertechnetate uptake (*P* < 0.001), and TT3 (*P* = 0.048) prior to RIT were found significantly associated with the outcomes of RIT by using the Mann-Whitney *U* test (Table 2). No statistical associations were found between post-RIT thyroid function and the following parameters: age at diagnosis, disease duration, FT4, FT3, and TT4. And no statistically significant association was found between post-RIT thyroid function (euthyroidism and hypothyroidism versus hyperthyroidism) and gender (*P* = 0.937) or ophthalmopathy (*P* = 0.395) by using the chi-squared test.

### 3.3. Thyroid Mass

**Figure 1:**
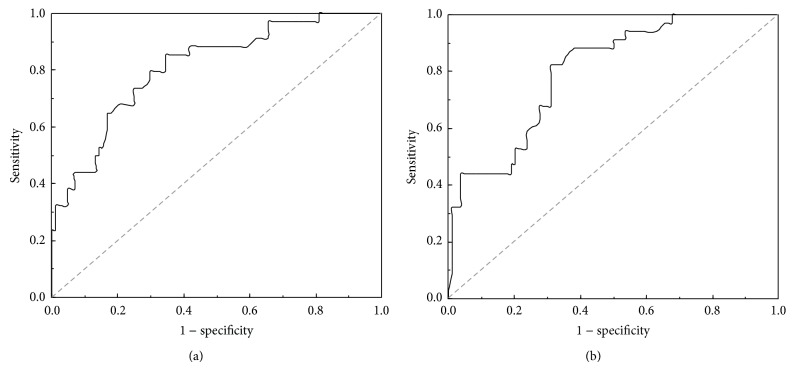
ROC curve used to identify cut-off values related to RIT success in patients with GD. (a) Thyroid mass. The area under the curve (AUC) was 0.811. The cut-off point of thyroid mass for prediction was estimated at 40.1 g, with 85.3% sensitivity and 65.5% specificity. (b) ^99m^Tc-pertechnetate uptake. AUC was 0.801. The cut-off point was estimated at 15.2%, with 82.4% sensitivity and 69.0% specificity.

We analyzed the estimated thyroid mass by using the ROC analysis to differentiate the patients who achieved treatment success (euthyroidism or hypothyroidism) from those who remained hyperthyroid (treatment failure) and found that patients of treatment success had a thyroid mass threshold of 40.1 g, with sensitivity of 85.3% and specificity of 65.5% (Figure 1(a)).

### 3.4. Thyroid ^99m^Tc-Pertechnetate Uptake

ROC analysis was used to analyze the ^99m^Tc-pertechnetate uptake obtained prior to radioiodine therapy to differentiate the group of patients who achieved success with treatment (euthyroidism and hypothyroidism) from those who remained hyperthyroid (treatment failure). We found treatment success patients had an uptake threshold of 15.2%, with sensitivity of 82.4% and specificity of 69.0% (Figure 1(b)).

### 3.5. Logistic Regression Analysis

Univariate logistic regression analysis showed statistical differences when comparing RIT success (euthyroidism and hypothyroidism) with failure (hyperthyroidism) to thyroid mass (*P* < 0.001) and ^99m^Tc-pertechnetate uptake (*P* < 0.001). There was no influence of gender (*P* = 0.939), ophthalmopathy (*P* = 0.395), age at diagnosis (*P* = 0.487), disease duration (*P* = 0.535), FT4 (*P* = 0.366), FT3 (*P* = 0.199), TT4 (*P* = 0.269), and TT3 (*P* = 0.118).

The multivariate logistic regression analysis demonstrated that the patients with thyroid mass ≤ 40.1 g showed a 6.35-fold higher probability of RIT success (odds ratio (OR) = 6.35, *P* = 0.002, 95% CI = 1.99–20.99) and patients with ^99m^Tc-pertechnetate uptake ≤ 15.2% presented a 4.77-fold higher probability of success (OR = 4.77, *P* = 0.007, 95% CI = 1.53–14.91). Kendall's correlation coefficient between thyroid mass and ^99m^Tc-pertechnetate uptake was 0.426 (*P* < 0.001).

## 4. Discussion

In the present study, GD patients received RIT with a single fixed dose of radioiodine (5 mCi). After RIT, 71.19% of the GD patients succeed (euthyroidism or hypothyroidism), but 25.51% of the patients still remained hyperthyroid. Moreover, patients with thyroid mass ≤ 40.1 g (odds ratio (OR) = 6.35) or ^99m^Tc-pertechnetate uptake ≤ 15.2% (OR = 4.77) had higher treatment success. However, we did not find a satisfied correlation coefficient between thyroid mass and ^99m^Tc-pertechnetate uptake (*r* = 0.426), which indicated a relative independence between the two predictive variables for thyroid function after RIT.

Although RIT for the treatment of GD has been used since the 1940s, there remains controversy about the optimal dosage of radioiodine. Theoretically, the RIT success rate increases with radioiodine dose, but incidence of hypothyroidism might also elevate, which might need a life-long hormone-replacement medication causing inconvenience to daily life. Often fixed activities of 370 or 555 MBq (10 or 15 mCi) are used, which will result in hypothyroidism in 69% to 90% of patients [4–7]. Fear of hypothyroidism also is the main reason for rejecting RIT as the first-line treatment for many GD patients in China [8]. In the present study, a fixed comparatively low dose radioiodine (5 mCi) was used with only 29.66% of GD patients becoming hypothyroid after RIT, but the overall RIT success (euthyroidism or hypothyroidism) is similar to the other previous studies [6, 7, 9, 10].

Thyroid scintigraphy is a conventional nuclear medicine procedure which provides valuable information regarding both thyroid anatomy and physiology and plays an integral role in the diagnosis and management of GD [11]. Thyroid scintigraphy using ^99m^Tc-pertechnetate has been proven to be more advantageous than with radioiodine, since the images have better quality, the procedure is faster, and the patient is submitted to a lower radiation dose [3]. As in just one visit, the patient receives an intravenous injection of ^99m^Tc-pertechnetate, the thyroid mass and ^99m^Tc-pertechnetate uptake can be measured after 20 min, and the radioiodide can be administered immediately afterwards. In the present study, we found that thyroid mass and ^99m^Tc-pertechnetate can be used to predict the outcomes of RIT. The results of this study will be very helpful in optimizing the therapeutical dose of radioiodine for GD patient, receiving a satisfactory rate of remission but providing the lowest possible radiation to the rest of the body.

In conclusion, in the present study, we found a fixed dose of 5 mCi radioiodine seems to be practical and effective, especially for the GD patients with thyroid mass ≤ 40.1 g and ^99m^Tc-pertechnetate uptake ≤ 15.2%. For GD patients larger goiter (> 40.1 g) and markedly increased ^99m^Tc-pertechnetate uptake (> 15.2%) prior to RIT can be considered as important predictive factors of RIT failure. These patients should be better candidates for receiving higher radioiodine doses or to be referred for thyroidectomy. Thus, ^99m^Tc-pertechnetate thyroid scintigraphy is an important prognostic factor for predicting the outcomes of RIT and it might also be helpful in optimizing the radioiodine dose of RIT.

## Figures and Tables

**Table 1:** Pre-RIT laboratory and clinical characteristics of patients.

Variables	Values
Gender	
Male	41 (34.75%)
Female	77 (65.25%)
Age (years)	40.35 ± 12.69 (17–77)
Disease duration (years)	4.20 ± 4.80 (0.08–30)
Ophthalmopathy	
Yes	73 (61.86%)
No	45 (38.14%)
Hormone levels of pre-RIT	
FT4 (pmol/L)	55.63 ± 27.19 (22.71–140.08)
FT3 (pmol/L)	19.99 ± 7.36 (7.46–44.39)
TT4 (nmol/L)	260.62 ± 80.07 (16.7–387.31)
TT3 (nmol/L)	7.08 ± 2.99 (1.01–12.32)
Thyroid mass of pre-RIT (g)	41.13 ± 8.85 (22.68–61.60)
Thyroid ^99m^Tc-pertechnetate uptake of pre-RIT (%)	15.23 ± 7.59 (2.80–36.80)
Outcome of post-RIT	
Hypothyroidism	35 (29.66%)
Euthyroidism	49 (41.53%)
Hyperthyroidism	34 (28.81%)

All values are expressed as mean ± SD for continuous variables and as the number of patients (percentage) for categorical variables.

**Table 2:** Comparative analysis on pre-RIT variables in different groups.

Variables	Patients group with different outcome of RIT		*P*
	Hypothyroidism and euthyroidism (*n* = 84)	Hyperthyroidism (*n* = 34)	
Age (years)	39.83 ± 12.88	41.62 ± 12.32	0.377
Disease duration (years)	4.37 ± 5.27	3.77 ± 3.40	0.631
Hormone levels			
FT4 (pmol/L)	54.20 ± 26.26	59.17 ± 29.47	0.463
FT3 (pmol/L)	19.44 ± 7.43	21.35 ± 7.09	0.152
TT4 (nmol/L)	255.46 ± 77.06	273.37 ± 86.95	0.470
TT3 (nmol/L)	6.80 ± 2.92	7.75 ± 3.09	0.048
Thyroid mass (g)	38.28 ± 7.34	48.18 ± 8.41	<0.001
^99m^Tc-pertechnetate uptake (%)	12.83 ± 6.05	21.19 ± 7.80	<0.001
